# Personal

**Published:** 1901-04

**Authors:** 


### PERSONAL.

DR. EUGENE A. SMITH, adjunct professor of clinical surgery at the University of Buffalo, spoke on the subject "Ideals of medicine," at the Lawyers' Club dinner, held at The Genesee, March 7, 1901. In the course of a very clever speech he said that there were two great ideals, the prevention of disease and the curing of disease. He spoke of personal hygiene in which purity of personal life, observance of a moral standard, plays so important a part. Municipal hygiene, with sanitation, also was discussed He told of what scientific medicine has done, instancing the conquering of diphtheria. He cited the great advances in medicine and surgery, of the good fortunes of the poor with hospitals affording them better treatment than the rich had fifty years ago. He traced disease by heredity, environment and habits, touched on the failures in medicine, referred to christian science, magnetic healing and osteopathy, then spoke of quackery and finally of ethics.

---

DR. DANIEL LEWIS, of New York, was recently appointed by the Governor to be State Health Commissioner and he was promptly confirmed by the senate. Dr. Lewis was president of the State Board of Health which has been legislated out of office, for three terms, and he has served as a member of the board since. His appointment as State Health Commissioner is for the term ending on December 31, 1904. The salary of the office is $3,500 a year. He will have entire jurisdiction over the matters which have heretofore been supervised by the State Board of Health.

Dr. Nelson W. Wilson, of Buffalo, has been appointed sanitary officer of the Pan-American exposition. The appointment emanates from Dr. Roswell Park, as medical director of the exposition, and is a fitting recognition of Dr. Wilson's ability in the direction named.

---

Maj. Charles K. Winne, Surgeon, U.S. Army, now at Fort Porter, was recently directed to report in person to the surgeon-general of the army, in order to consult in regard to the exhibit of the medical department of the army to be made at the Pan-American exposition.

---

Dr. Matthew D. Mann, of Buffalo, recently addressed the students of the Masten Park High School, taking for his subject, The medical profession. The address was in the nature of advice to students who are thinking of entering the profession of medicine.

---

Dr. Charles W. Farr, formerly of Buffalo, has been appointed assistant surgeon in the United States army, with the rank of first lieutenant. Dr. Farr has lately returned from the Philippines, where he has been serving as contract surgeon.

---

Dr. J. Glen Ernest has removed from Buffalo to Gasport, Niagara County, N.Y. Dr. Ernest is one of the rising younger members of the profession of medicine, and will prove an undoubted gain to the people in his new environment.

---

Dr. W. J. O'Donnell, of Buffalo, announces the removal of his office from 399 Front Ave., to 269 Carolina Street, cor. West Avenue and Tracy Street, March 1, 1901. Hours, 9 to 10 a.m.; 1 to 3 and 7 p.m.

---

Dr. James P. G. Gould, of Buffalo, U. of B. '99, is now pursuing post-graduate studies in New York, but expects to return about May 1, 1901. He will then establish himself on Franklin street.

---

Dr. Ernest Wende, health commissioner of Buffalo, read a paper by invitation before the Chicago Medical Association, Wednesday evening, March 6, 1901, on The miscarriage of municipal sanitation.

---

Dr. B. A. Gipple, of Alden, N.Y., was elected a member of the board of supervisors of Erie County last month. Dr. Gipple, we believe is the only physician who will sit in the new board.

Dr. Edgar A. Forsyth, of Buffalo, has removed from 348 Franklin Street, to 326 Franklin Street. His practice is limited to diseases of the nose, throat and ear. Hours: 9 to 1 and 7 to 8.

Dr. Edmond E. Blaauw, of Buffalo, N.Y., has been appointed editor of Dutch ophthalmic literature in the *Annals of Ophthalmology*, in the place of Dr. Wendell Reber, resigned.

Dr. Vertner Kenerson, of Buffalo, has been appointed assistant medical director of the Pan-American Exposition and will have immediate charge of the ambulance service.

Dr. Eugene Wasdin of the Marine Hospital Service, delivered two lectures on Yellow fever in Alumni Hall, at the University of Buffalo, March 11 and 18, 1901, from 5 to 6 p.m.

Dr. Helene Kuhlmann, assistant physician at the Buffalo State Hospital, has gone to Johns Hopkins University, where she will spend six weeks in study.

Dr. Nelson G. Russell, of Buffalo, has been appointed assistant surgeon 65th Regiment, N. Y. N. G., vice Captain Harry Mead, M. D., resigned.

Dr. Ira C. Brown, of Buffalo, has been appointed a surgeon of volunteers, with the rank of major. Dr. Brown is still serving in the Philippines.

Dr. James E. King, of Buffalo, U. of B. '96, recently sailed for Europe, where he will spend six months in professional study, chiefly of obstetrics and gynecology.

Dr. Arthur G. Bennett, of Buffalo, announces his removal from 191 Delaware Avenue to 26 Allen Street. Hours: 9 to 1.

Dr. J. Henry Dowd, of Buffalo, will remove May 1, 1901, from 288 Franklin Street to the Cutler, 380 Franklin Street.

